# Environment-Associated Variations in Blood Metabolism of Mongolian Cattle Grazing in the Alxa Desert of China

**DOI:** 10.3390/vetsci12050506

**Published:** 2025-05-21

**Authors:** Chao Hai, Dongchao Pei, Yuqing Yang, Lishuang Song, Xuefei Liu, Chunling Bai, Guanghua Su, Lei Yang, Guangpeng Li

**Affiliations:** State Key Laboratory of Reproductive Regulation and Breeding of Grassland Livestock, College of Life Science, Inner Mongolia University, Hohhot 010070, China; h15248037201@163.com (C.H.); pdc102035@163.com (D.P.); yyq284219588@163.com (Y.Y.); xiaoshuang2000@126.com (L.S.); liuxuefei1006@126.com (X.L.); chunling1980_0@163.com (C.B.); suguanghua0707@163.com (G.S.)

**Keywords:** Mongolian cattle, desert environment, serum biochemical indices, blood metabolism

## Abstract

Desert environments pose severe challenges to livestock survival. This study examined climate-driven physiological and metabolic adaptations in 258 Mongolian cattle from six regions of the Alxa Desert, China. Serum biochemical indices were measured and analyzed using linear models to assess the effects of climate, sex, and age. Climate significantly affected key blood parameters, including glucose (*p* < 0.001), creatinine (*p* < 0.001), alkaline phosphatase (*p* < 0.001), and lactate (*p* = 0.034). Additionally, sex significantly influenced lactate dehydrogenase (*p* = 0.049), bicarbonate (*p* = 0.0061), urea (*p* = 0.0055), and triglycerides (*p* = 0.039), while age affected total protein (*p* = 0.020), LDL-C (*p* = 0.0097), and cholesterol (*p* < 0.001). Glucose levels were negatively correlated with body size traits. Metabolomic profiling showed that cattle in arid, high-radiation areas exhibited reduced TCA cycle and fatty acid metabolism, with concurrent carbohydrate accumulation, including glucose, fructose, and mannose. Enhanced amino acid metabolism increased proline, valine, tyrosine, and tryptophan levels, potentially supporting physiological stability under heat and drought stress. These findings reveal how Mongolian cattle modulate metabolism in response to desert climates, offering insights into livestock adaptation and informing breeding strategies for resilience in harsh environments.

## Simple Summary

Alxa Mongolian cattle live in desert regions with extreme heat, strong sunlight, and little rainfall. To survive in this harsh environment, they have developed special ways to adapt. In this study, we examined their blood and metabolism and found that changes in glucose, calcium, and amino acids help them cope with heat and dryness. Their body size, hormone levels, and energy use also support survival in the desert. These results help us better understand how animals adapt to climate stress and may help improve livestock breeding in tough environments.

## 1. Introduction

Deserts are among the most extreme terrestrial ecosystems on Earth, characterized by chronic water scarcity, high temperatures, intense solar radiation, and low humidity. They occupy approximately one-third of the global land surface and host the largest terrestrial biome by area [1]. These harsh conditions have exerted strong selective pressures on the organisms inhabiting them, shaping unique physiological and genetic adaptations over long evolutionary periods. Consequently, a stable relationship has developed between species’ genetic makeup and their climatic environment. However, with the increasing impact of climate change, shifts in environmental parameters are expected to alter genetic expression and reduce genetic diversity across populations [2].

Water scarcity remains the most critical stressor in arid zones, but additional climatic factors—such as prolonged sunlight exposure and high ambient temperatures—exacerbate physiological stress in desert fauna [3]. Heat stress arises when animals are unable to effectively dissipate accumulated body heat due to extreme environmental conditions [4]. As a result, biodiversity in deserts is often lower than in more temperate ecosystems. Nevertheless, many desert-adapted animals exhibit a suite of complex responses, including physiological acclimation, behavioral adaptation, genetic variation, and long-term evolutionary modifications [5,6].

Modern breeding programs typically prioritize productivity traits under optimal temperature and humidity conditions, often within controlled environments. However, this strategy may compromise animals’ resilience and limit their adaptability to increasingly variable and extreme climates [7]. Mongolian cattle, distributed in regions of Mongolia and northern China—including Inner Mongolia—are renowned for their adaptability, disease resistance, and capacity to thrive in resource-scarce environments. In particular, populations grazing in the Badain Jaran and Tengger Deserts—both part of the Alxa Desert in northwest China—represent a unique model for studying natural adaptation to desert ecosystems. Despite their biological importance, the mechanisms underlying their physiological adaptation to extreme climates remain poorly understood.

In this study, we investigated 258 naturally grazing Mongolian cattle from six distinct sites within the Alxa Desert to elucidate the relationship between climatic conditions and blood traits. By analyzing hematological parameters, serum biochemical profiles, and metabolic signatures, we aimed to reveal the physiological and biochemical strategies that enable Mongolian cattle to survive and thrive in desert environments. This work contributes to a deeper understanding of mammalian adaptation to extreme climates and offers insights into the conservation of adaptive genetic resources critical for future livestock resilience.

## 2. Materials and Methods

### 2.1. Animals and Sample Sites

**Figure 1:**
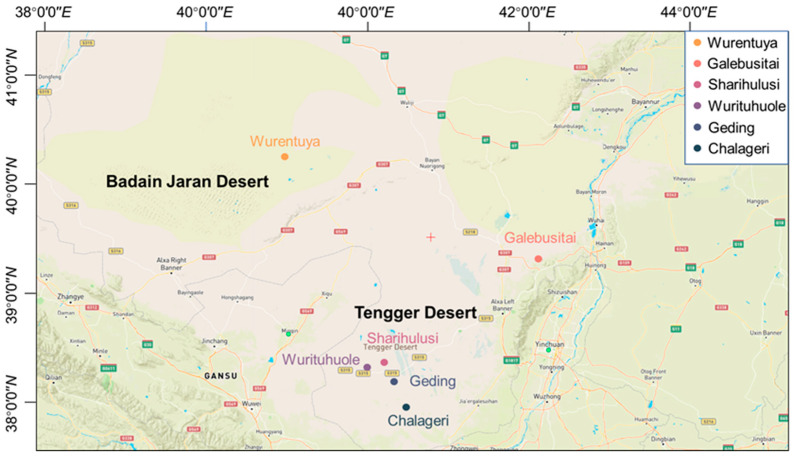
Map showing 6 sampling sites of Mongolian cattle in Alxa Desert. The map was edited and generated with Meteonorm software v8.0.2.

A total of 258 Mongolian cattle, including 40 males and 218 females, were selected for this study. All animals graze year-round on natural desert pastures in the Alxa League, Inner Mongolia Autonomous Region. The cattle were randomly chosen from a pool of healthy, disease-free individuals and are considered representative of the local indigenous breed. Animals were herded from open desert grazing areas into temporary enclosures for measurement and blood sampling. Six sampling locations were selected, spanning diverse desert microenvironments: Wurentuya (40.3° N/103.0° E), Galebusitai (39.3° N/106.1° E), Sharihulusi (38.4° N/104.2° E), Wurituhuole (38.3° N/104.0° E), Geding (38.2° N/104.4° E), and Chalageri (37.9° N/104.5° E) (Figure 1).

**Figure 2:**
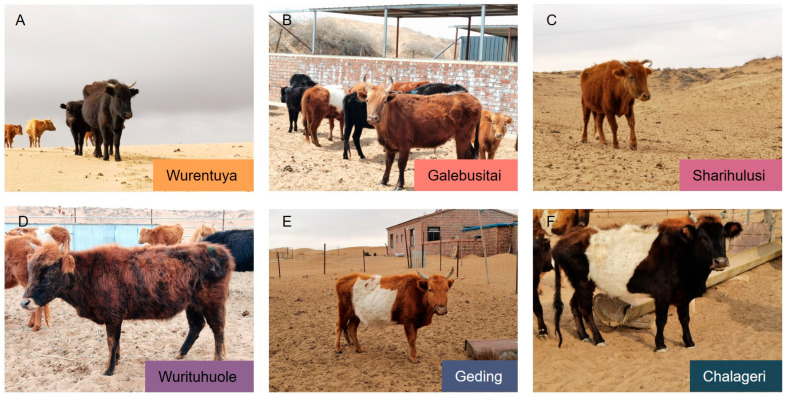
Representative Mongolian cattle from six sampling sites in the Alxa Desert. This figure displays the phenotypic diversity of Mongolian cattle naturally grazing in six distinct desert environments within the Alxa region.

The sampling was conducted in spring, a period characterized by extremely dry conditions and minimal forage availability across the Badain Jaran and Tengger Deserts. These regions exhibit a typical arid desert climate, marked by high solar radiation, low precipitation (annual average < 100 mm), large diurnal temperature variations, and prolonged sunshine hours, which together create a harsh and water-limited environment. No antibiotics or concentrated feed were administered at any stage of the animals’ lives, ensuring that physiological parameters reflect natural adaptations. The age of each individual was determined through local farm records. Due to varied genetic backgrounds, coat colors of the Mongolian cattle ranged from yellow and light yellow to brown and mixed tones (Figure 2).

### 2.2. Developmental Index

The Mongolian cattle were grouped together and measured for BW and body size; then, we collected blood. For specific methods, please refer to our previous report [8]. BW, body length and width were measured using a scale (kilograms) and ruler (cm). All measurements were taken by the same experienced researcher to ensure consistency and accuracy.

### 2.3. Detection of Blood Physiological and Biochemical Indexes

Cattle blood samples were collected from sampling localities for hematological and biochemical analyses. Of these, approximately 231 blood samples were collected in tubes containing ethylenediaminetetraacetic acid (EDTA) as an anticoagulant for hematological analysis, while 258 blood samples were collected in serum separator tubes (SSTs) for biochemical analysis. Hematological examinations were performed within 24 h of blood collection. The collected blood samples were then analyzed by ABX PENTRA 80 (HORIBA ABX SAS, Montpellier, France) to determine the monocytes (MONs), allogeneic lymphocytes (ALYs), red blood cells (RBCs), hematocrit (HCT), hemoglobin (HGB), large immature cells (LICs), red cell distribution width (RDW), mean corpuscular hemoglobin (MCH), mean corpuscular volume (MCV), mean corpuscular hemoglobin concentration (MCHC), mean platelet volume (MPV), basophilic granulocyte (BAS), white blood cells (WBCs), plateletcrit (PCT), platelet (PLT), and lymphocytes (LYMs). Plasma was separated from 258 blood samples by centrifugation at 3000 rpm for 10 min at room temperature and stored at 4 °C to estimate biochemical parameters until further analysis. Biochemical indexes of glucose (Glu), alanine aminotransferase (ALT), aspartate aminotransferase (AST), total protein (TP), albumin (ALB), creatine kinase (CK), high-density lipoprotein cholesterol (HDL_C), low-density lipoprotein cholesterol (LDL_C), amylase (AMY), calcium (Ca), alkaline phosphatase (ALP), lactate dehydrogenase (LDH_L), bicarbonate (CO2_L), cholinesterase (CHE), cholesterol (CHOL), lactic acid (LACT), triglyceride (TRIGL), urea (UREAL), lipase (LIPC), and creatinine (CRE) were determined using the Cobas c311 analyzer (ROCHE).

### 2.4. Climate Data

Meteonorm is a powerful climate data generation software with a robust climate database. The software is capable of using interpolation methods to extract climate data for every selected station near the desired location. It can generate data on an hourly or even minute basis. Therefore, in this study, Meteonorm 8.0.2 was used to generate climate data. Six main climatic components, including Ta, Sd, RH, RR, GHI and DHI, were used to investigate the environmental adaptability of Mongolian cattle in desert areas. Wurentuya (uncertainty of yearly values: GHI = 9%, Ta = 1.5 °C; radiation interpolation locations: share of satellite data: 100%), Galebusitai (uncertainty of yearly values: GHI = 7%, Ta = 1.1 °C; radiation interpolation locations: Yinchuan (95 km), Yijinhuoluoqi (317 km), Minqin (273 km), Dongsheng (336 km); share of satellite data: 72%); temperature interpolation locations: Yinchuan (95 km), Minqin (273 km)), Sharihulusi (38.4 °N/104.2 °E), Wurituhuole (uncertainty of yearly values: GHI = 9%, Ta = 1.1 °C; radiation interpolation locations: share of satellite data: 100%; temperature interpolation locations: Minqin (90 km), Yinchuan (190 km), Zhangye (320 km)), Geding (uncertainty of yearly values: GHI = 9%, Ta = 1.5 °C; radiation interpolation locations: share of satellite data: 100%; temperature interpolation locations: Minqin (122 km), Yinchuan (165 km), Zhangye (351 km), Yulin (466 km)) and Chalageri (uncertainty of yearly values: GHI = 9%, Ta = 1.5 °C; radiation interpolation locations: share of satellite data: 100%; temperature interpolation locations: Minqin (146 km), Yinchuan (160 km), Lanzhou (218 km)). The monthly data statistics are based on the average data from each day for the full 24 h.

### 2.5. Untargeted Metabolomic Study

A total of 20 serum samples were collected from Wurentuya and Galebusitai and subsequently divided into four groups. Additionally, five serum samples from each of the remaining four locations (Sharihulusi, Wurituhuole, Geding, and Chalageri) were pooled to form one composite group, resulting in a total of four experimental groups. A high-resolution tandem mass spectrometer, TripleTOF5600plus (SCIEX, Macclesfield, UK), was used to detect serum metabolites eluted form the column. The Q-TOF was operated in both positive and negative ion modes. The curtain gas was set at 30 PSI, ion source gas1 was set at 60 PSI, ion source gas2 was set 60 PSI, and an interface heater was used, the temperature of which was 650 °C. The acquired MS data pretreatments, including peak picking, peak grouping, retention time correction, second peak grouping, and annotation of isotopes and adducts, were performed using XCMS software (v3.7.1). LC/MS raw data files were converted into mzXML format and then processed by the XCMS, CAMERA and metaX toolbox implemented with the R software. Each ion was identified by combining retention time (RT) and *m*/*z* data.

The online KEGG HMDB database was used to annotate the metabolites by matching the exact molecular mass data (*m*/*z*) of samples with those from the database. If a mass difference between observed and the database value was less than 10 ppm, the metabolite would be annotated and the molecular formula of metabolites would further be identified and validated by the isotopic distribution measurements.

The intensity of peak data was further preprocessed by an in-house software, metaX (v2.0.0.007 https://github.com/arisnetxsolutions/METAX-2.0/releases/tag/2.0.0.007 accessed on 18 May 2025). Features that were detected in less than 50% of QC samples or 80% of biological samples were removed, and the remaining peaks with missing values were imputed with the k-nearest neighbor algorithm to further improve the data quality.

### 2.6. Data Analysis

To evaluate the effects of sex, age, and sampling site on the serum biochemical indices of Mongolian cattle, a multiple linear regression model was applied using R software (v3.4.1). In this model, each biochemical index was treated as a dependent variable, while sex, age, and site were included as independent variables. This approach allowed us to quantitatively assess the effect of sex, age, and site on biochemical traits.

The multiple linear regression used for the analysis of serum biochemical index was as follows:

(1) y=b0+b1i+b2j+b3k+ε

where y = the response variables (Glu, AST, ALT, TP, ALB, CK, HDL-C, LDL-C, AMY, Ca, ALP, LDH, CO2-L, CHE, CHOL, LACT, TRIGL, UREA, LIPC, CRE); b0 = the intercept in multivariate linear regression; b1i = effect of sex (female, male); b2j = effect of age (1–14 years old); b3k = effect of sites (Wurentuya, Galebusitai, Sharihulusi, Wurituhuole, Geding and Chalageri). ε = the random error.

Statistical analysis and mapping of climate data was performed using GraphPad Prism v8.3.0. All datasets were initially subjected to Kolmogorov–Smirnov’s test for homogeneity of variance (Table S1a,e,i,m,q). As the number of days varied from month to month, analysis of a mixed model was used for multiple comparisons for two variables, sites and mouth, because repeated measures ANOVA cannot handle missing values. Partial Least Squares Discriminant (PLS-DA) and principal component analysis (PCA) were performed with R v4.2.1. Redundancy analysis (RDA) was performed using the OmicStudio tools (available online: https://www.omicstudio.cn/tool/ accessed on 18 May 2025). Pathway analysis of differential metabolites was conducted using MetaboAnalyst 5.0 (available online: https://www.metaboanalyst.ca/ accessed on 18 May 2025) [9]. A significance level of 0.05 was used for all statistical tests.

## 3. Results

### 3.1. Spatial Variations in Desert Climate Across Mongolian Cattle Sampling Sites

**Figure 3:**
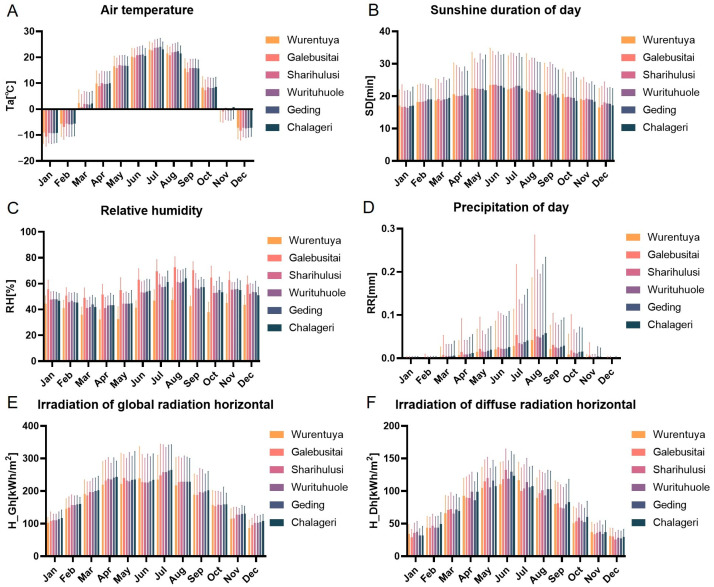
Different mouths of climate index of six sampling sites. (**A**–**F**) Statistical analysis of air temperature (Ta), sunshine duration (SD) in hours, relative humidity (RH), precipitation (RR) in hours, and irradiation in terms of global horizontal radiation (H_Gh) and diffuse horizontal radiation (H_Dh).

To investigate the environmental conditions potentially influencing the adaptability of Mongolian cattle, we analyzed six key climatic variables at the six desert sampling sites in the Alxa region: air temperature (Ta), sunshine duration (Sd), relative humidity (RH), precipitation (RR), global horizontal irradiance (GHI), and diffuse horizontal irradiance (DHI). The data revealed substantial spatial heterogeneity in climatic conditions across the sites (Figure 3, Table S1b,f,j,n,r).

Wurentuya, located deep within the Badain Jaran Desert, recorded the lowest annual air temperatures, while Sharihulusi, Wurituhuole, Geding, and Chalageri, situated further south, exhibited the highest temperatures—though differences among these warmer sites were not statistically significant.

Sd showed a clear latitudinal gradient: higher-latitude sites, such as Wurentuya, experienced longer daylight hours throughout the year, whereas Chalageri, at a lower latitude, had the shortest duration.

Rh and RR patterns further distinguished the sites. Galebusitai, positioned on the fringe of the Tengger Desert, had the highest relative humidity and precipitation, while Wurentuya had the lowest. Although monthly RR values showed minimal variation in February, the other months reflected significant differences between sites (Figure 3C,D, Table S1k,l).

The GHI was significantly higher at lower-latitude sites, particularly Sharihulusi, Wurituhuole, Geding, and Chalageri, compared to Wurentuya. The DHI exhibited a similar latitudinal trend but without significant inter-site differences.

Taken together, these climatic data suggest a broad environmental gradient within the Alxa Desert region. Sites such as Sharihulusi, Wurituhuole, Geding, and Chalageri in the central Tengger Desert share similar climatic profiles, while Galebusitai (Tengger Desert edge) and Wurentuya (deep Badain Jaran Desert) represent environmental extremes, with notably different temperature, radiation, and moisture conditions.

### 3.2. Analysis of Blood Routine and Biochemistry

**Figure 4:**
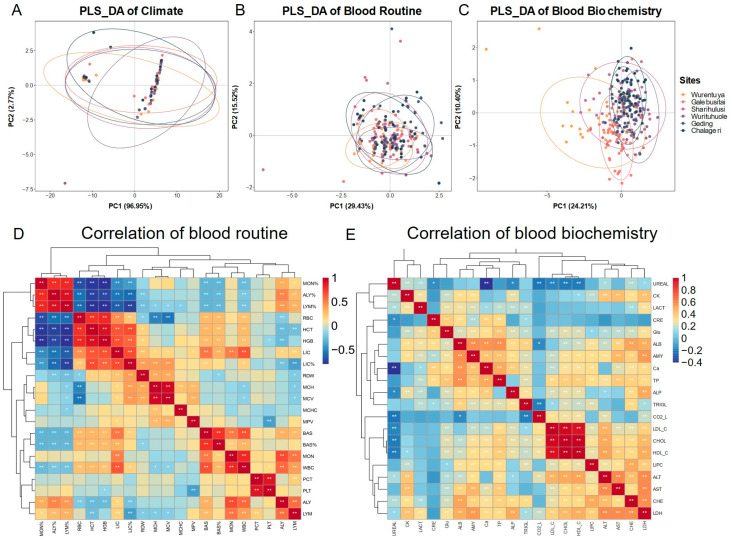
Partial Least Squares- Discriminant Analysis (PLS-DA) of climate, blood routine and biochemistry. (**A**) PLS_DA plot of January–December average climate parameters for different regions. (**B**) PLS_DA plot of 231 blood routine for different regions. (**C**) PLS_DA plot of 258 blood biochemistry for different regions. (**D**) Correlation of blood routine. (**E**) Correlation of serum biochemistry. Asterisks indicate statistically significant differences, * *p*  <  0.05; ** *p*  <  0.01.

In harsh desert environments, animals often adapt by modifying their blood physiological and biochemical parameters. To explore the impact of environmental diversity on Mongolian cattle, we performed Partial Least Squares Discriminant Analysis (PLS-DA) on climatic parameters, blood routine indicators (*n* = 231), and serum biochemical indicators (*n* = 258) from different sampling locations (Figure 4).

The PLS-DA of climate parameters (Figure 4A) revealed that the annual climatic conditions across desert regions were generally similar, with only slight variation in monthly patterns. Most sampling sites clustered tightly together, suggesting a consistent desert climate. However, distinct outliers reflected the unique climatic characteristics of specific sites.

PLS-DA of blood routine indicators (Figure 4B) showed that most cattle clustered closely, indicating similar hematological profiles across sites. However, individuals from Geding and Chalageri exhibited greater variability. Interestingly, cattle from the relatively humid Galebusitai site had lower white blood cell (WBC) counts, including lymphocytes (LYMs) and monocytes (MONs), compared to drier regions. Conversely, in the driest region, Wurentuya, red blood cell (RBC) counts and hematocrit (HCT) were reduced, while red blood cell morphology parameters such as mean corpuscular volume (MCV) and red cell distribution width (RDW) were elevated. Mean corpuscular hemoglobin concentration (MCHC) remained stable across regions, averaging around 28.78.

Serum biochemistry (Figure 4C) revealed greater sensitivity to environmental variation. Cattle from Wurentuya and Galebusitai displayed distinct biochemical profiles, with Wurentuya cattle nearly completely separated from others. Elevated liver function markers (ALT, AST) were found in Galebusitai cattle, likely linked to the relatively humid conditions, with average levels exceeding 61.28 ± 17.49 (AST) and 22.14 ± 7.52 (ALT). In contrast, these indicators were lowest in Wurentuya. Blood urea nitrogen (UREAL), reflecting kidney function, was significantly lower in Wurentuya (0.88 ± 1.27) than the overall average (3.6 ± 2.3), suggesting potential dehydration-related adaptations. Overall, these results indicate that environmental differences across desert regions have a stronger influence on serum biochemical profiles than on routine blood parameters.

Correlation analysis further revealed patterns of physiological adaptation. Among the blood routine indicators (Figure 4D), strong positive correlations were observed between MON%, ALY%, and LYM%, and between RBC, HCT, hemoglobin (HGB), and large immature cells (LICs, LIC%). These two clusters of indicators were negatively correlated with each other. BAS, BAS%, MON, and WBC were positively associated, while plateletcrit (PCT) correlated strongly with platelet count (PLT).

In contrast, correlations among serum biochemical indicators were generally weaker (Figure 4E). However, strong positive correlations were evident among cholesterol (CHOL), low-density lipoprotein cholesterol (LDL_C), and high-density lipoprotein cholesterol (HDL_C). Additionally, ALT, AST, cholinesterase (CHE), and lactate dehydrogenase (LDH) showed relatively strong mutual correlations, suggesting coordinated liver metabolic activity.

### 3.3. Influence of Climate on Blood Routine and Biochemistry

To evaluate the physiological adaptation of Alxa Mongolian cattle to diverse desert environmental conditions, we constructed a redundancy analysis (RDA) model to explore the relationship between climatic variables and both blood routine and biochemical parameters.

The RDA results indicated that several blood routine parameters, such as hemoglobin (HGB) and hematocrit (HCT), tend to increase under conditions of dehydration caused by limited water availability. As the body attempts to compensate for water loss, hemoconcentration occurs, elevating these parameters. Additionally, biochemical markers such as serum creatinine (CRE) and various electrolytes were also influenced by dehydration. Elevated creatinine levels may result from the kidney’s effort to conserve water by concentrating urine. Electrolyte imbalances, including hyponatremia, hyperkalemia, and hypercalcemia, were also observed and are likely linked to disruptions in fluid balance.

**Figure 5:**
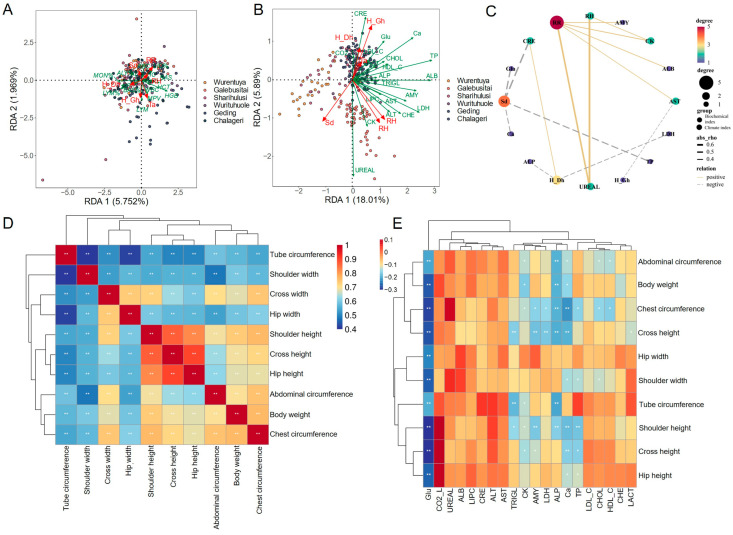
A redundancy analysis (RDA) between climate index and blood index. (**A**) RDA of blood routine and climate index; (**B**) RDA of serum biochemistry and climate index; (**C**) correlation of blood biochemistry and climate index; (**D**) correlation of growth parameters; (**E**) correlation of blood biochemistry and growth parameters. Asterisks indicate statistically significant differences, * *p*  <  0.05; ** *p*  <  0.01.

Extreme temperatures and environmental stress may further stimulate the release of stress-related hormones such as cortisol and epinephrine, which are known to alter blood glucose (Glu) levels and could potentially increase the risk of metabolic syndrome or diabetes. Higher ambient temperatures can also elevate enzymatic activity and metabolic rates, thereby enhancing oxygen delivery to tissues and contributing to increased HGB concentrations. Prolonged sunlight exposure has been associated with a reduction in white blood cell (WBC) count, which may impair immune function. Furthermore, long-term exposure to non-ionizing radiation may reduce both red blood cell (RBC) counts and hemoglobin concentrations, potentially leading to immune suppression, blood pressure fluctuations, and oxidative stress (Figure 5A).

Compared to hematological parameters, blood biochemistry showed more pronounced differences across sampling sites (Figure 5B). Mongolian cattle from Sharihulusi, Wurituhuole, Geding, and Chalegeri—areas characterized by high air temperatures, intense solar radiation, and arid conditions—exhibited significant elevations in most biochemical indices. In contrast, Galebusitai cattle, which inhabit regions with longer daylight hours and higher relative humidity, showed elevated levels of urea (UREAL) and creatine kinase (CK), with values located between Sharihulusi and RH in the RDA plot.

Interestingly, in the high-radiation and high-temperature areas (Sharihulusi, Wurituhuole, Geding, and Chalegeri), serum creatinine (CRE) was consistently elevated and strongly associated with climatic indices such as air temperature (Ta), drought index (DHI), and heat index (GHI) (Figure 5B,C).

To further investigate environmental effects on animal development, we analyzed the growth traits of all sampled Mongolian cattle. Due to limited biological resources and sparse vegetation in the desert, the average body weight was relatively low, at 187.75 ± 49.34 kg. In the harshest environments—characterized by high temperature and radiation—cattle exhibited the lowest body weights and reduced growth performance (Table S2).

Correlation analysis revealed that all measured growth parameters were positively associated with body weight, with correlation coefficients exceeding 40%. Chest and abdominal girths showed the strongest correlations, followed by height-related traits, while width-related traits had the weakest correlations (Figure 5D). Notably, blood Glu levels were negatively correlated with growth parameters, suggesting that elevated stress-induced glucose may adversely affect growth. Conversely, UREAL, which positively correlates with relative humidity (RH) and respiratory rate (RR), also showed a positive correlation with body size, indicating that relatively humid environments may promote better growth in Alxa Mongolian cattle (Figure 5E).

Together, these findings highlight the strong influence of desert climatic conditions on both physiological and developmental traits of Alxa Mongolian cattle.

### 3.4. Multiple Linear Regression Analysis of Biochemical Parameters

Upon detailed analysis, it was observed that the relationship between blood routine parameters and climatic variables was less pronounced than that between serum biochemical parameters and climate. Given the geographic proximity and similar environmental conditions among the sampling sites of Sharihulusi, Wurituhuole, Geding, and Chalageri, we grouped them under a unified designation—Tengger Desert Center (TDC)—to represent a region characterized by high temperatures and intense solar radiation.

To identify the climatic variables with the greatest impact on serum biochemistry, we employed multiple linear regression analysis using gender, age, and climatic conditions as independent variables. The analysis revealed that several serum biochemical indicators, including glucose (Glu), aspartate aminotransferase (AST), creatine kinase (CK), alkaline phosphatase (ALP), lactate dehydrogenase (LDH), lactic acid (LACT), and creatinine (CRE), were significantly influenced by climate (*p* < 0.05). Among these, blood glucose (Glu) exhibited the most significant climate-related effect (*p* = 8.29 × 10^−7^) (Table 1 and Table S2).

In addition to climate, the effects of sex and age on serum biochemical parameters were also assessed. The results showed that sex significantly influenced LDH (*p* = 0.049), bicarbonate (CO2-L) (*p* = 0.0061), UREAL (*p* = 0.0055) and TRIGL (*p* = 0.039). Age had significant effects on total protein (TP) (*p* = 0.020), low-density lipoprotein cholesterol (LDL-C) (*p* = 0.0097), amylase (AMY) (*p* = 0.0238), ALP (*p* = 0.0009)*,* cholesterol (CHOL) (*p* < 0.001), and urea (UREAL) (*p* = 0.0154).

**Figure 6:**
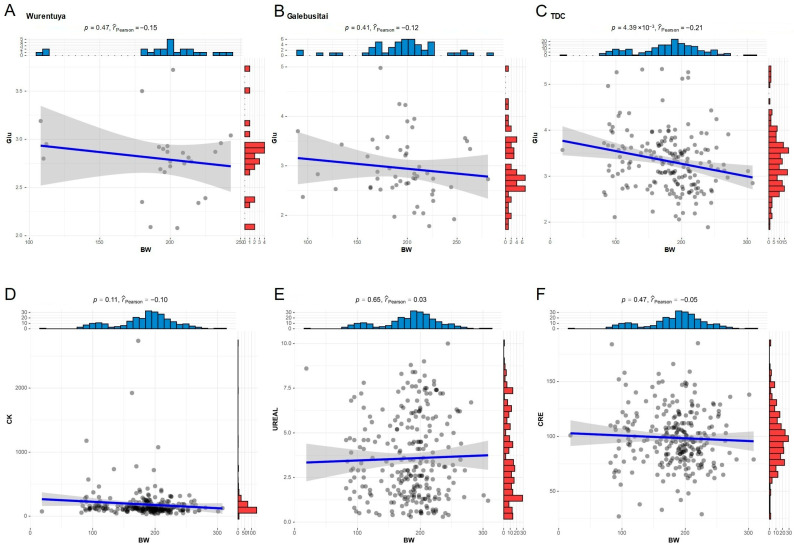
Relationship between blood Glu and BW under different climatic conditions. (**A**–**C**) Correlation between BW and blood Glu in cattle sampled at different climatic sites; (**D**–**F**) correlation of CK, UREAL and CRE with BW.

In light of earlier findings demonstrating a strong negative correlation between blood Glu and various growth metrics, we selected body weight (BW) as a representative marker for growth and development to further assess this relationship. The analysis showed a significant negative correlation between BW and blood Glu specifically within the arid and high-radiation TDC region, with the strongest negative correlation detected in this area (Figure 6A–C). These results suggest that elevated blood glucose levels, potentially driven by climatic stressors, may hinder growth in Mongolian cattle.

In contrast, other climate-associated biochemical parameters such as CK, UREAL, and CRE did not exhibit a significant impact on growth traits (Figure 6D,E). This implies that although these indicators reflect physiological stress or metabolic adjustments in response to environmental conditions, they are not directly predictive of growth performance in desert-adapted Mongolian cattle.

### 3.5. Blood Metabolome of Mongolian Cattle

**Figure 7:**
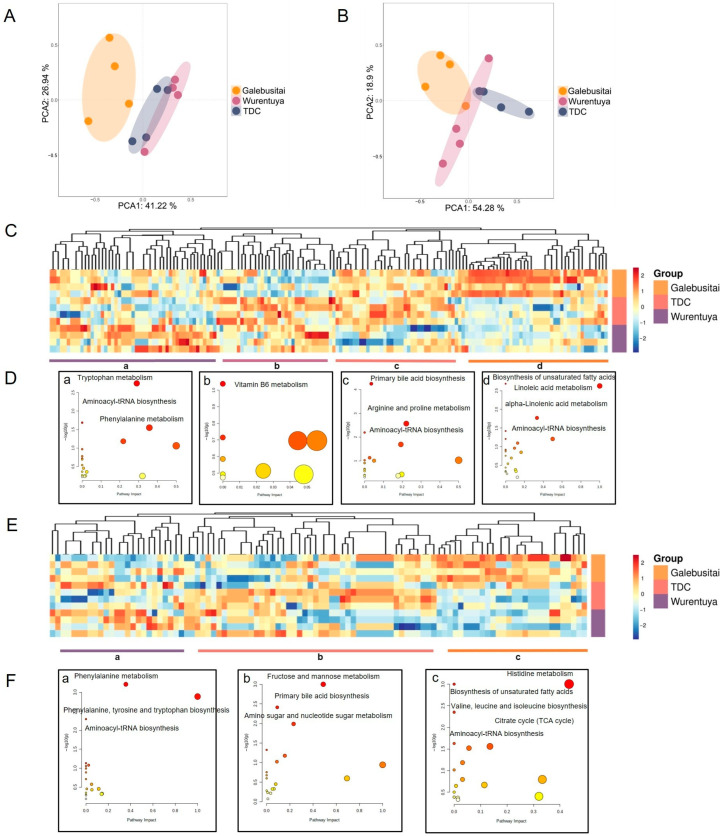
Serum metabolome of Mongolian cattle in the Alxa Desert. (**A**,**B**) Principal component analysis (PCA) of the serum metabolome in (**A**) positive and (**B**) negative ion mode for different climate groups. (**C**,**E**) Heatmap of Mongolian cattle serum metabolites from Wurentuya, Tengger Desert Centre (TDC), and Galebusitai. Relative abundances were log-transformed and normalized with a z-score method. (**D**,**F**) KEGG pathway enrichment of serum metabolites from different climate environments. The small letters in (**D**) and (**F**) correspond to the labeled metabolites in heatmaps (**C**) and (**E**), respectively.

To further elucidate the mechanisms underlying the adaptability of Mongolian cattle to desert environments, we performed blood metabolomics analysis across different climatic regions. Principal component analysis (PCA) of blood metabolites, conducted in both positive and negative ion modes, revealed distinct metabolic profiles among cattle from the four climate zones. Specifically, the metabolomic profile of cattle from Galebusitai differed markedly from those of the TDC and Wurentuya groups (Figure 7A,B).

Subsequent clustering analysis of the metabolites identified in the positive-ion mode revealed four distinct groups, representing elevated metabolite abundance in the following: (1) Galebusitai; (2) Galebusitai and TDC; (3) TDC and Wurentuya; and (4) Wurentuya (Figure 7C). In the negative-ion mode, metabolites were classified into three groups, corresponding to higher abundance in Galebusitai, TDC, and Wurentuya, respectively (Figure 7E).

To interpret the functional significance of these differences, we conducted KEGG pathway enrichment analysis on the highly abundant metabolites in each group (Figure 7D,F). Despite regional differences, several overlapping pathways were enriched across sites—most notably, aminoacyl-tRNA biosynthesis—reflecting the complex metabolic adaptations required to withstand environmental stressors such as food and water scarcity, extreme heat, and prolonged drought conditions.

Compared with the relatively humid Galebusitai region, the drier regions (TDC and Wurentuya) exhibited distinct metabolic shifts. These included a marked depletion of tricarboxylic acid (TCA) cycle intermediates such as isocitric acid and citric acid (Figure 7(F-a)), a reduction in unsaturated fatty acid metabolism, and a significant accumulation of carbohydrates including fructose, mannose, and glucose (consistent with serum biochemical findings, Figure 7(D-d,F-b,c)).

Moreover, there was enhanced activity in amino acid metabolic pathways—particularly arginine and proline metabolism and tryptophan metabolism—resulting in elevated levels of several amino acids, such as proline, serine, and valine (Figure 7A,(F-a)). These findings suggest that under desert stress conditions, Alxa Mongolian cattle shift their metabolic strategies by promoting glycolysis and amino acid biosynthesis, while suppressing TCA cycle activity. This reprogramming may help conserve energy and water, and increase the synthesis of stress-related metabolites such as proline, phenylalanine, tyrosine, and tryptophan.

Overall, drought stress appears to drive a metabolic transition characterized by enhanced carbohydrate and amino acid metabolism, alongside inhibited oxidative phosphorylation. This reflects an adaptive response aimed at maintaining physiological homeostasis and supporting survival in arid desert environments.

## 4. Discussion

Climate change, particularly warming temperatures and altered precipitation patterns, is reshaping ecosystems and influencing the physiological adaptability of many animals, including mammals [10,11,12]. Birds and mammals often adapt behaviorally, physiologically, and morphologically to cope with environmental fluctuations [13,14], yet studies focusing on mammalian adaptation to climate change remain limited [15]. In this study, we focused on Alxa Mongolian cattle living in a typical desert climate characterized by intense solar radiation, minimal precipitation, and extreme diurnal temperature variation. These environmental pressures significantly influence blood physiological traits, providing potential evidence of adaptive responses.

To assess adaptability, we selected six desert sites to capture climatic variation across regions and analyzed blood parameters from all available Mongolian cattle. A larger sample size increases the reliability and precision of regression models [16], and although cattle numbers varied across regions, the random nature of these differences likely minimizes bias [17,18].

Environmental stress, particularly heat stress, alters nutrient intake, rumination, and endocrine function, contributing to energy imbalance and reduced productivity [6]. In goats and sheep, heat stress leads to a decreased metabolic rate, increased sweating, and modified respiratory and hormonal activities [19,20,21]. Cattle primarily rely on skin and respiratory pathways for cooling [22], and while species like pigs and birds adopt different strategies due to anatomical differences [23,24,25], mammals such as Mongolian cattle possess sweat glands that aid thermoregulation in arid climates [26].

Furthermore, desert environments with high solar radiation and temperature extremes can impair gut microbial diversity [27], contributing to reduced nutrient absorption and metabolic efficiency. Body size is also crucial for heat adaptation—smaller body mass helps reduce metabolic heat production. We found that adult Alxa Mongolian cattle averaged at only ~200 kg, similarly to other heat-adapted species like Amazonian birds [28,29].

Physiological responses were reflected in blood biochemical indicators. Sunlight duration negatively correlated with glucose and calcium levels (Figure 5), aligning with previous findings that sunlight affects glucose metabolism [30,31] and calcium absorption via vitamin D [32]. Elevated blood calcium may adversely affect growth (Figure 5D). Amino acids such as leucine and isoleucine modulate glucose levels via different mechanisms, with leucine stimulating insulin and glutaminolysis, while isoleucine promotes glucose uptake and reduces gluconeogenesis [33,34,35,36,37,38,39].

Sex is known to influence numerous metabolic pathways due to hormonal and physiological differences, leading to observable variations in serum biochemical indicators between males and females [40]. Lactate dehydrogenase is a key enzyme in aerobic glycolysis, catalyzing the conversion of pyruvate into lactate and playing an important role in metabolism [41]. The higher LDH levels observed in bulls (Table 1 and Table S2) may be attributed to their greater muscle mass and higher physical activity, which demands enhanced glycolytic metabolism and the increased activity of muscle-associated enzymes [42,43]. In contrast, cows exhibited higher serum urea levels. This difference may reflect the fact that in bulls, a larger proportion of dietary protein is utilized for muscle synthesis rather than degradation, resulting in lower urea production [44]. The elevated metabolic activity in bulls is also supported by their higher bicarbonate, indicating stronger buffering capacity and better acid–base regulation [45]. This may be related to the greater metabolic rate and respiratory activity typically observed in male cattle [46,47]. Additionally, the higher triglyceride levels in bulls may reflect differences in lipid metabolism, which could be influenced by hormonal regulation, such as testosterone [48]. Testosterone is known to promote lipid mobilization and storage during growth, which may explain the observed sex-based metabolic differences [49].

Drought-related stress triggered widespread metabolic changes in Alxa Mongolian cattle, affecting pathways such as aminoacyl-tRNA biosynthesis, the TCA cycle, gluconeogenesis, and proline synthesis (Figure 7), indicative of increased energy demand and biosynthetic activity under environmental duress [50,51,52], possibly due to dehydration and intense radiation altering gene expression and metabolic regulation [50,53].

## 5. Conclusions

This study demonstrates that Alxa Mongolian cattle have developed distinct physiological and metabolic adaptations to survive in harsh desert environments characterized by high temperatures, intense solar radiation, and limited water availability. Alterations in blood biochemical indicators, including glucose, calcium, and amino acids, along with changes in key metabolic pathways, suggest a coordinated response to environmental stress. These findings provide valuable insights into the adaptive mechanisms of indigenous cattle breeds and highlight potential biomarkers for selecting climate-resilient livestock. Understanding such adaptive traits is critical for developing sustainable breeding strategies in the context of global climate change.

## Figures and Tables

**Table 1:** Significance analysis of sex, age, and climate on biochemical parameters.

Parameters	*p*-Value		
	Sex	Age	Climate
Glu (mmol/L)	0.498	0.715	8.29 × 10^−7^ ***
AST (U/L)	0.697	0.249	0.0212 *
ALT (U/L)	0.721	0.085	0.785
TP (g/L)	0.867	0.020 *	0.806
ALB (g/L)	0.273	0.524	0.502
CK (U/L)	0.126	0.073	0.0334 *
HDL-C (mmol/L)	0.709	0.126	0.827
LDL-C (mmol/L)	0.685	0.0097 **	0.374
AMY (U/L)	0.209	0.0238 *	0.641
Ca (mmol/l)	0.855	0.872	0.955
ALP (U/L)	0.195	0.000947 ***	0.000510 ***
LDH (U/L)	0.0490 *	0.228	0.0134 *
CO2-L (mmol/L)	0.00612 **	0.00231 **	0.293
CHE (U/L)	0.928	0.572	0.174
CHOL (mmol/L)	0.776	0.000874 ***	0.487
LACT (mmol/L)	0.302	0.383	0.03391 *
TRIGL (mmol/L)	0.03918 *	0.439	0.461
UREAL (mmol/L)	0.00551 **	0.01537 *	0.610
LIPC (U/L)	0.371	0.908	0.095
CRE (μmol/L)	0.819	0.274	4.84 × 10^−6^ ***

Asterisks indicate statistically significant differences (* *p*  <  0.05; ** *p*  <  0.01; *** *p*  <  0.001).

## Data Availability

The original contributions presented in the study are included in the article/Supplementary Materials; further inquiries can be directed to the corresponding author/s.

## Supplementary Materials

The following supporting information can be downloaded at https://www.mdpi.com/article/10.3390/vetsci12050506/s1: Table S1: Statistical analysis of climate data; Table S2: Blood routine, biochemistry and growth parameters data of Mongolian cattle.
